# KCNQ2 channels regulate the population activity of neonatal GABAergic neurons *ex vivo*

**DOI:** 10.3389/fneur.2023.1207539

**Published:** 2023-06-20

**Authors:** Bowen Hou, Sabato Santaniello, Anastasios V. Tzingounis

**Affiliations:** ^1^Department of Physiology and Neurobiology, University of Connecticut, Storrs, CT, United States; ^2^Department of Biomedical Engineering and CT Institute for the Brain and Cognitive Sciences, University of Connecticut, Storrs, CT, United States

**Keywords:** KCNQ2, epilepsy, neurological disorders, interneurons, channelopathy, neonate

## Abstract

Over the last decade KCNQ2 channels have arisen as fundamental and indispensable regulators of neonatal brain excitability, with KCNQ2 loss-of-function pathogenic variants being increasingly identified in patients with developmental and epileptic encephalopathy. However, the mechanisms by which KCNQ2 loss-of-function variants lead to network dysfunction are not fully known. An important remaining knowledge gap is whether loss of KCNQ2 function alters GABAergic interneuron activity early in development. To address this question, we applied mesoscale calcium imaging *ex vivo* in postnatal day 4–7 mice lacking KCNQ2 channels in interneurons (*Vgat-ires-cre;Kcnq2^f/f^;GCamp5*). In the presence of elevated extracellular potassium concentrations, ablation of KCNQ2 channels from GABAergic cells increased the interneuron population activity in the hippocampal formation and regions of the neocortex. We found that this increased population activity depends on fast synaptic transmission, with excitatory transmission promoting the activity and GABAergic transmission curtailing it. Together, our data show that loss of function of KCNQ2 channels from interneurons increases the network excitability of the immature GABAergic circuits, revealing a new function of KCNQ2 channels in interneuron physiology in the developing brain.

## Introduction

Pathogenic variants in multiple potassium channels have been associated with neurodevelopmental disorders, including epilepsy and autism spectrum disorders (1–3). In particular, members of the KCNQ (Kv7) channel family have been linked to pediatric epilepsy disorders ranging from benign self-limiting epilepsy to severe forms like developmental and epileptic encephalopathy (DEE) (4–6). The KCNQ family is a subdivision of the voltage-gated potassium channel superfamily and is comprised of five members, KCNQ1 to KCNQ5 (Kv7.1-Kv7.5), named based on the order of identification (7–9). Three of the five members (KCNQ2, KCNQ3, and KCNQ5) have been implicated in epilepsy disorders, with KCNQ2 channels being the most frequently identified member (10). Indeed, KCNQ2 variants are the most common genetic causes of early onset neonatal epilepsy (5).

The KCNQ2 channel was first identified in 1998 as a gene underlying benign familial neonatal convulsions (11, 12), now renamed as self-limited (familial) neonatal epilepsy (SLFNE). As the name suggests, seizures resolve within a short period of time without looming cognitive issues later in life, although a small percentage of these patients have an increased propensity for future epilepsy (13). In addition to SLFNE, pathogenic KCNQ2 variants have been identified in patients with DEE (4, 14). These variants are predominantly loss-of-function and act in a dominant-negative fashion, leading to seizures shortly after birth (4, 5). Although the seizures can resolve over time, these patients have persistent developmental issues such as intellectual disability, impaired communication abilities, sleep disruption, and a disrupted ability to feed themselves (15, 16). Currently, the mechanisms that underlie the majority of the symptoms associated with KCNQ2 DEE are unclear (4).

Unlike the majority of voltage-gated potassium channels, KCNQ2 channels are expressed early in the nervous system, prior to birth (17, 18). For instance, a study found that KCNQ2 channels are expressed in humans starting at the 20th midgestational week (19). Similarly, KCNQ2 protein can be clearly detected in embryonic cells and at birth in mice (18, 20). Following birth, KCNQ2 protein levels steadily increase (21, 22), partly due to the progressive downregulation of microRNAs targeting KCNQ2 mRNA and preventing its translation early in development (22). Consistent with the presence of KCNQ2 expression early in development, we and others have found that deletion of KCNQ2 channels or expression of loss of function KCNQ2 variants in forebrain neurons leads to profound changes in hyperexcitability across the neocortex and hippocampus in the first week of life (23, 24). Consistent with this finding, application of pan-KCNQ2 blockers increased neuronal activity and bursting in slices prepared from one-week-old animals (25). These changes in excitability are due to the loss of KCNQ2 channels mediating the M-current, medium afterhyperpolarization, and in some cell types the slow afterhyperpolarization (26–28). Together, these potassium currents act as a brake to excessive firing activity in excitatory cells, counteracting the effects of the persistent sodium current, a subthreshold conductance that increases neuronal activity (29–31).

Although KCNQ2 channels are associated with the M-current and spike frequency adaptation, a feature most commonly associated with excitatory glutamatergic cells, KCNQ2 channel expression is not restricted to excitatory cells, as it is also found in GABAergic interneurons (32–35). Indeed, we previously found that deleting *Kcnq2* from parvalbumin interneurons elevated their excitability, thus increasing the frequency of spontaneous inhibitory synaptic potentials in pyramidal neurons (35, 36). Similarly, studies using knockin mice expressing *Kcnq2* pathogenic variants (p.Tyr284Cys; pThr274Met) across all cell types also reported increased GABAergic synaptic activity in the hippocampus (34) and neocortex (24). In addition to genetic perturbations, pharmacologically inhibiting or activating KCNQ channels can increase or dampen interneuron excitability, respectively, supporting the notion that KCNQ channels regulate interneuron firing properties (33, 35, 36).

With few exceptions (34), past studies on KCNQ channels and interneuron excitability have taken place in juvenile or adult neurons. Considering the early expression of KCNQ2 channels in the brain (17, 18), here we examined whether deleting *Kcnq2* solely from GABAergic interneurons alters interneuron network excitability in the neonatal forebrain. We utilized mesoscale calcium imaging in order to monitor the effect of KCNQ2 protein loss from GABAergic interneurons across the neocortex and hippocampus in parallel (23). We found that *Kcnq2* deletion leads to increased GABAergic interneuron network excitability, suggesting that KCNQ2 loss-of-function pathogenic variants would alter not only excitatory neuronal excitability, but also GABAergic interneuron network excitability early in development, an effect that might contribute to the developmental and epileptic encephalopathy phenotype of KCNQ2 DEE patients.

## Materials and methods

The animal study was reviewed and approved by the University of Connecticut institutional animal care and use committee (IACUC).

All mice were kept on a C57BL/6 J background. The *Kcnq2^f/f^* mice have been previously described and published (23, 37, 38). To express Cre recombinase in GABAergic cells, we used the *Slc32a1^tm2(cre)/Lowl^/J* mouse line, which drives Cre recombinase from the *Slc32a1* promoter region (RRID:IMSR_JAX:016962). *Slc32a1^tm2(cre)/Lowl^/J* mice are also known as Vgat-ires-cre mice; hereafter, we refer to these mice as VGAT-cre. To detect calcium events in neurons, we used B6;129S6-Polr2a^Tn(pb-CAG-GCaMP5g,-tdTomato)Tvrd^/J mice (RRID:IMSR_JAX:024477|PC-G5-tdT), which express Gcamp5g in the presence of Cre recombinase (39). This enabled the expression of GCamp5g as well as dtTomato when Cre recombinase was present. The expression of dtTomato allowed us to determine the extent of Cre recombinase activity and the specificity, ensuring no ectopic expression. Hereafter, we refer to the interneuron-specific *Kcnq2* knockout mice as IN:Kcnq2. Breeding was performed at the University of Connecticut Vivarium. For this study, we developed and used *VGAT-cre::PC-G5-tdT::Kcnq2^f/+^* mice. We intercrossed these mice to obtain *VGAT-cre::PC-G5-tdT::Kcnq2^+/+^*, *VGAT-cre::PC-G5-tdT::Kcnq2^f/+^*, and *VGAT-cre::PC-G5-tdT::Kcnq2^f/f^* mice. For our experiments, we used either *VGAT-cre::PC-G5-tdT::Kcnq2^+/+^* or *VGAT-cre::PC-G5-tdT::Kcnq2^f/f^* mice. Genotypes were confirmed with PCR before each experiment. Experiments were conducted without considering sex preference as there have been no reported effects of sex-dependency on KCNQ2 patients (40). Mice had food and water *ad libitum* and were kept in a 12:12 light/dark cycle.

### Acute brain slice preparation

P4–P7 mice were anesthetized using isoflurane. Anesthetized mice were then decapitated rapidly and their brains were quickly dissected out. The removed brains were transferred into an ice-cold cutting solution containing: 26 mm NaHCO_3_, 210 mm sucrose, 10 mm glucose, 2.5 mm KCl, 1.25 mm NaH_2_PO_4_, 0.5 mm CaCl_2_, and 7 mm MgCl_2_. The brains were kept in a holding chamber for ~1 min prior to relocation to a vibratome (Leica, VS2000) for slicing. Slices, 300 μm in thickness, were obtained and transferred into an incubation chamber warmed to 35–37°C. Slices were kept at 35–37°C for 30 min. After that, the incubation container and the slices were placed on a benchtop at room temperature for at least 1 hour prior to experiments. The incubation chamber contained artificial cerebrospinal fluid (ACSF) with the following components: 125 mM NaCl, 26 mM NaHCO_3_, 2.5 mM KCl, 1 mM NaH_2_PO_4_, 1.3 mM MgCl_2_, 1.5 mM CaCl_2_, and 12 mM d-glucose.

### Acute brain slice imaging

To image calcium activity, slices were placed in a Warner holding chamber superfused continuously with warm (33–35°C) ACSF. For recording calcium activity, we used an Axio Zoom V.16 (Zeiss) microscope with a PlanNeoFluar Z 1X, 0.25NA objective and a 56 mm working distance. Using multi-imaging acquisition software (Micromanager), 300 s long movies recorded using an sCMOS camera (pco.edge 4.2) at a 500 × 513 pixel resolution (50 ms exposure time; 6,000 frames).

### Analysis of calcium events

TIFF files (6,000 frames per file) were imported into the Fiji-ImageJ^1^ (41) software package. For each TIFF file, we selected a region of interest (ROI) such as the CA3 region of the hippocampus or the anterior, medial, or posterior cortex to measure changes in calcium fluorescence. Using the Fiji-ImageJ built-in functions, we plotted the *z*-axis profile for each ROI, and then obtained the fluorescent value per each frame for every ROI. The fluorescent values (*F*) for the 300 s-long recording period were then exported to Axograph analysis software (v.1.7.5, https://axograph.com/). Using the built-in functions of Axograph, we obtained the Δ*F*/*F* time series for each calcium event. The cut-off point for a recorded calcium event was 0.01 Δ*F*/*F*. For each calcium event, we measured its amplitude and duration (50% of the peak).

ROIs and signals Δ*F*/*F* for the 2D plots were determined according to a semi-automatic procedure as described in (23). Briefly, frames (500 × 500 pixels, inter-pixel distance: 6 μm) were inspected for the external edges of the area covered by each hemisphere, and only pixels within the area delineated by these lines were further considered. Pixels within this area were divided into ROIs by using the SLIC (simple linear iterative clustering) algorithm (42) and, for each ROI, the fluorescence signal *F* was estimated as the average value of the fluorescence intensity across all pixels in the ROI. The series of variations Δ*F* was then estimated for each ROI as Δ*F* = (*F* − *m*)/*m*, where *m* is the moving average value of *F* (moving window: 30 s). ROIs were numbered consecutively along the anteroposterior direction, with lower values assigned to ROIs towards the anterior cortex and higher values assigned to ROIs towards the posterior cortex and the CA3 area of the hippocampus.

### Local field potential recording and analysis

In parallel to calcium fluorescence events, we recorded the local field potentials (LFP) from the CA3 region of the hippocampus using a microarray with eight bipolar electrodes (MicroProbes). Data were recorded through a RHD2000 multichannel amplifier with RHD2000 interface software (v1.5, Intan Technologies) at a 20 kHz sample rate and imported in MATLAB (R2020b, MathWorks, Inc.). The raw data were lowpass filtered (20^th^ order FIR filter, cutoff frequency 1.3 kHz), and the resultant signals were further filtered (cutoff frequency: 80 Hz, 8^th^ order Chebyshev Type I lowpass filter applied in both directions to avoid phase distortions) before being down-sampled to 200 Hz. For each recording, 30 s-long segments were selected for spectral analyses. LFP channels in each segment were standardized and processed via continuous wavelet transform (10 voices per octave, analytic bump wavelet) to perform a time-frequency analysis. All analyses were performed blind to the genotype.

### Statistics

GraphPad Prism 8 software (GraphPad) was used to graph the data for statistical analyses. We used Mann–Whitney U tests to determine significance between groups of data.

## Results

### Loss of KCNQ2 channels from GABAergic cells primarily impacts network activity in the hippocampal formation

**Figure 1:**
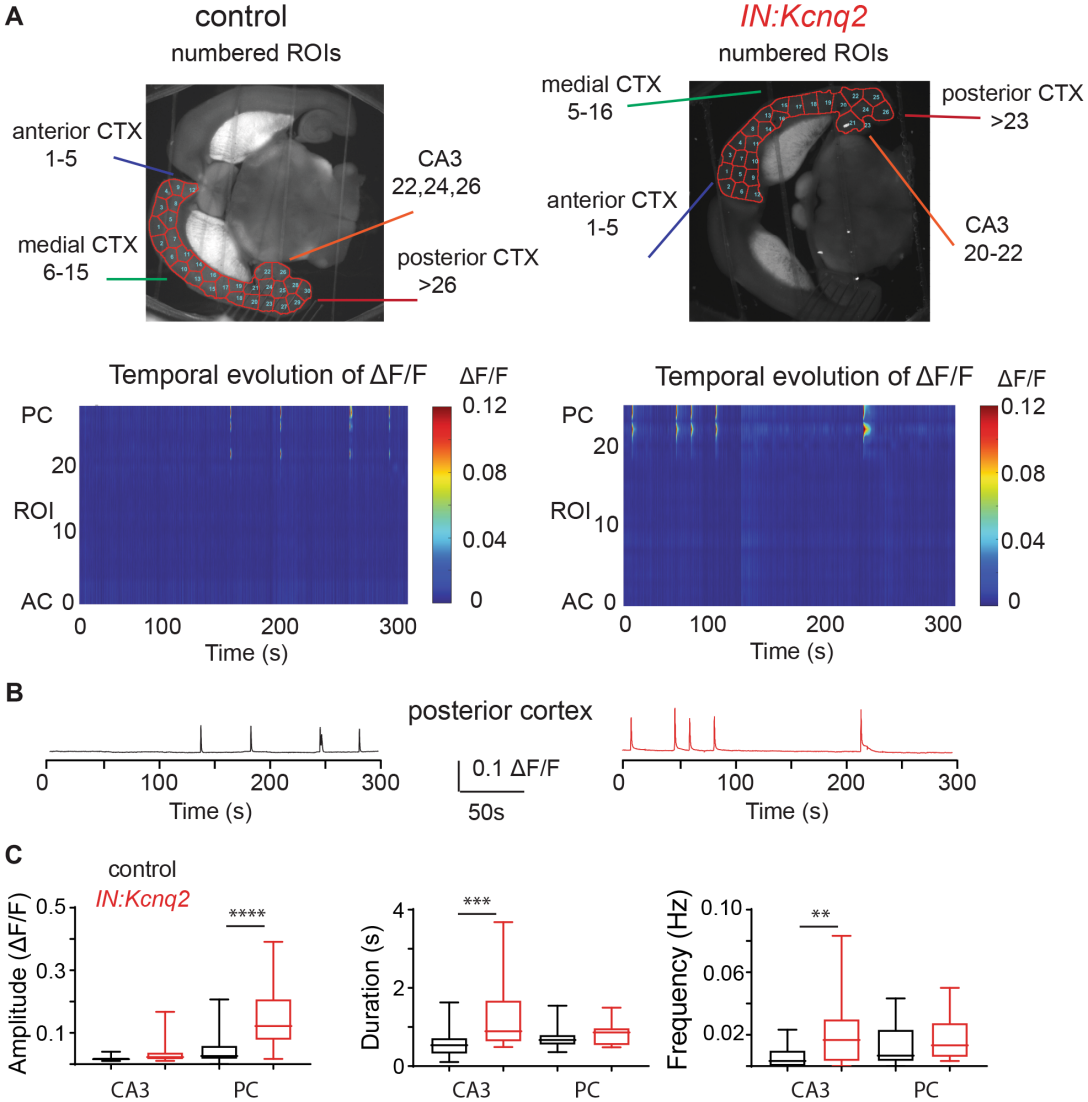
*Kcnq2* deletion from neonatal GABAergic interneurons elevates hippocampal formation and posterior cortex excitability. **(A)** Top panels: representative examples of horizontals slices expressing GCamp5g from control and *IN:Kcnq2* mice. Each hemisphere was segmented into regions of interest (ROI). Bottom panels: 2D plots showing the changes in the calcium signal measured as Δ*F*/*F* for the different ROIs during the 5 min recording period. The ROI numbering represents the various ROIs with lower values towards the anterior cortex and higher values towards the posterior cortex and the CA3 area of the hippocampus. **(B)** Representative example of change in calcium fluorescence (Δ*F*/*F*) observed in the posterior cortex. The data are from the slice depicted in **(A)**. **(C)** Summary graphs showing the effect of *Kcnq2* deletion to the amplitude (Δ*F*/*F*), duration (s), and number of events per second (Hz) for ROIs representing the CA3 area of the hippocampus and posterior cortex. Data are presented as box-and-whisker plots (box shows the 25th and 75th percentiles, whereas the whiskers show the minimum and maximum values). Detailed data and statistics for these graphs are reported under Table 1; Figure 1C section.

To determine whether KCNQ2 channels alter interneuron GABAergic network excitability, we first recorded interneuron activity under basal conditions (2.5 mM Ko). For these experiments, we used horizontal slices, preserving the connections between different network subregions. Figure 1 compares the fluorescence activity across different hemispheres. We note that the observed calcium fluorescence changes reflect activity across a population of neurons, as mesoscale imaging does not allow for detection of calcium events at a single-cell resolution. In our control slices, the regions with the highest basal activity were the hippocampal formation and in particular the CA3 region of the hippocampus and the posterior cortex, likely reflecting the entorhinal cortex (Figures 1A,B). This was consistent with earlier reports showing that the hippocampal formation shows spontaneous activity in slices from neonatal mice (43, 44).

**Table 1:** Summary data and statistical analysis for Figures 1–6.

Figure 1							
Figure			Control	*IN:Kcnq2*	Statistical tests and values		
**1C**	CA3	Amplit.	0.0194 ± 0.002 Δ*F*/*F*	0.0368 ± 0.008 Δ*F*/*F*	Mann–Whitney U test	*p* = 0.0631	Control: *n* = 17; *IN:Kcnq2*: *n* = 22 hemispheres
		Duration	0.5688 ± 0.090 s	1.256 ± 0.185 s	Mann–Whitney U test	*p* = 0.0006	Control: *n* = 17; *IN:Kcnq2*: *n* = 22 hemispheres
		Events/s	0.0062 ± 0.001 Hz	0.0214 ± 0.004 Hz	Mann–Whitney U test	*p* = 0.0021	Control: *n* = 28; *IN:Kcnq2*: *n* = 27 hemispheres
	Post. Cortex	Amplit.	0.0533 ± 0.011 Δ*F*/*F*	0.146 ± 0.019 Δ*F*/*F*	Mann–Whitney U test	*p* < 0.0001	Control: *n* = 24; *IN:Kcnq2*: *n* = 26 hemispheres
		Duration	1.15 ± 0.24 s	5.3 ± 0.61 s	Mann–Whitney U test	*p* = 0.3203	Control: *n* = 24; *IN:Kcnq2*: *n* = 26 hemispheres
		Events/s	0.008 ± 0.0018 Hz	0.015 ± 0.0018 Hz	Mann–Whitney U test	*p* = 0.0045	Control: *n* = 28; *IN:Kcnq2*: *n* = 26 hemispheres
							Animals: control: *n* = 6; *IN:Kcnq2*: *n* = 6.

Figure 2							
Figure			Control	*IN:Kcnq2*	Statistical tests and values		
**2C**	CA3	Amplit.	0.077 ± 0.004 Δ*F*/*F*	0.102 ± 0.009 Δ*F*/*F*	Mann–Whitney U test	*p* = 0.1289	Control: *n* = 63; *IN:Kcnq2*: *n* = 56 hemispheres
		Duration	0.916 ± 0.088 s	1.147 ± 0.083 s	Mann–Whitney U test	*p* = 0.0015	
		Events/s	0.311 ± 0.018 Hz	0.240 ± 0.017 Hz	Mann–Whitney U test	*p* = 0.0047	
	Post. Cortex (PC)	Amplit.	0.08 ± 0.006 Δ*F*/*F*	0.29 ± 0.023 Δ*F*/*F*	Mann–Whitney U test	*p* < 0.0001	Control: *n* = 67; *IN:Kcnq2*: *n* = 52 hemispheres
		Duration	2.46 ± 0.16 s	2.54 ± 0.15 s	Mann–Whitney U test	*p* = 0.4825	
		Events/s	0.150 ± 0.010 Hz	0.196 ± 0.013 Hz	Mann–Whitney U test	*p* = 0.0030	
	Medial Cortex (MC)	Amplit.	0.027 ± 0.003 Δ*F*/*F*	0.26 ± 0.003 Δ*F*/*F*	Mann–Whitney U test	*p* = 0.087	Control, n = 61; *IN:Kcnq2*: *n* = 50 hemispheres
		Duration	2.77 ± 0.46 s	5.58 ± 0.43 s	Mann–Whitney U test	*p* < 0.0001	
		Events/s	0.016 ± 0.003 Hz	0.043 ± 0.0076 Hz	Mann–Whitney U test	*p* = 0.0065	
	Anter. Cortex (AC)	Amplit.	0.037 ± 0.003 Δ*F*/*F*	0.065 ± 0.007 Δ*F*/*F*	Mann–Whitney U test	*p* < 0.0001	Control: *n* = 61; *IN:Kcnq2*: *n* = 47 hemispheres
		Duration	1.16 ± 0.13 s	7.02 ± 0.83 s	Mann–Whitney U test	*p* < 0.0001	
		Events/s	0.022 ± 0.0034 Hz	0.047 ± 0.006 Hz	Mann–Whitney U test	*p* = 0.022	
Data were obtained from 10 control and 8 *IN:Kcnq2 mice*.							

Figure 3						
Figure		Control	*IN:Kcnq2*	Statistical tests and values		
**3C**	1–5 Hz	16.24 ± 1.59 μV^2^/Hz^2^	16.83 ± 1.62 μV^2^/Hz^2^	Mann–Whitney U test	*p* = 0.073	Control: *n* = 14; *IN:Kcnq2*: *n* = 14 bursts
	5–10 Hz	21.94 ± 1.72 μV^2^/Hz^2^	26.80 ± 1.17 μV^2^/Hz^2^	Mann–Whitney U test	*p* = 0.0067	
	10–15 Hz	24.28 ± 2.48 μV^2^/Hz^2^	29.35 ± 2.26 μV^2^/Hz^2^	Mann–Whitney U test	*p* = 0.114	
	15–20 Hz	18.32 ± 2.59 μV^2^/Hz^2^	20.95 ± 1.94 μV^2^/Hz^2^	Mann–Whitney U test	*p* = 0.603	
	20+ Hz	70.89 ± 9.76 μV^2^/Hz^2^	89.27 ± 11.06 μV^2^/Hz^2^	Welch’s t test	*p* = 0.224	
Data were obtained from 3 control and 3 *IN:Kcnq2 mice*.						

Figure 4							
Figure			Control	*IN:Kcnq2*	Statistical tests and values		
**4C**	CA3	Amplit.	−PTX 0.095 Δ*F*/*F* ± 0.07 S.D.	−PTX 0.10 Δ*F*/*F* ± 0.08 S.D.	Mann–Whitney U test	Control *p* < 0.0001	Control: -PTX: *n* = 823; +PTX: *n* = 824 events
			+PTX 0.20 Δ*F*/*F* ± 0.13 S.D.	+PTX 0.22 Δ*F*/*F* ± 0.015 S.D.	Mann–Whitney U test	*IN:Kcnq2 p* < 0.0001	
		Duration	−PTX 0.76 s ± 1.2 S.D.	−PTX 0.92 s ± 1.4 S.D.	Mann–Whitney U test	Control *p* < 0.0001	
			+PTX 0.95 ± 1.2 S.D.	+PTX 1.6 ± 3.0 S.D.	Mann–Whitney U test	*IN:Kcnq2 p* < 0.0001	
							*IN:Kcnq2*: -PTX: *n* = 1,278 events; +PTX: *n* = 573 events
	Post. Cortex	Amplit.	−PTX 0.09 Δ*F*/*F* ± 0.08 S.D.	−PTX 0.13 Δ*F*/*F* ± 0.12 S.D.	Mann–Whitney U test	Control *p* < 0.0001	Control: -PTX: *n* = 483; +PTX: *n* = 533 events
			+PTX 0.25 Δ*F*/*F* ± 0.16 S.D.	+PTX 0.25 Δ*F*/*F* ± 0.22 S.D.	Mann–Whitney U test	*IN:Kcnq2 p* < 0.0001	
		Duration	−PTX 3.0 s ± 3.2 S.D.	−PTX 2.5 s ± 2.7 S.D.	Mann–Whitney U test	Control *p* < 0.0001	
			+PTX 2.2 s ± 3.7 S.D.	+PTX 2.1 ± 3.5 S.D.	Mann–Whitney U test	*IN:Kcnq2 p* < 0.0001	
							*IN:Kcnq2*: -PTX: *n* = 641; +PTX: *n* = 604 events
	Medial Cortex	Amplit.	−PTX 0.03 Δ*F*/*F* ± 0.04 S.D.	−PTX 0.02 Δ*F*/*F* ± 0.007 S.D.	Mann–Whitney U test	Control *p* < 0.0001	Control: -PTX: *n* = 91; +PTX: *n* = 200 events
			+PTX 0.15 Δ*F*/*F* ± 0.13 S.D.	+PTX 0.09 Δ*F*/*F* ± 0.12 S.D.	Mann–Whitney U test	*IN:Kcnq2 p* < 0.0001	
		Duration	−PTX 3.2 s ± 4.7 S.D.	−PTX 3.4 s ± 3.5 S.D.	Mann–Whitney U test	Control *p* < 0.0001	
			+PTX 2.2 s ± 7.3 S.D.	+PTX 3.9 ± 5.3 S.D.	Mann–Whitney U test	*IN:Kcnq2 p* = 0.0073	
							*IN:Kcnq2*: -PTX: *n* = 171; +PTX: *n* = 208 events
	Anter. Cortex	Amplit.	−PTX 0.041 Δ*F*/*F* ± 0.030S.D.	−PTX 0.06Δ*F*/*F* ± 0.05 S.D.	Mann–Whitney U test	Control: *p* < 0.0001	Control: -PTX: *n* = 74; +PTX: *n* = 204 events
			+PTX 0.13 Δ*F*/*F* ± 0.09 S.D.	+PTX 0.13 Δ*F*/*F* ± 0.1 S.D.	Mann–Whitney U test	*IN:Kcnq2 p* < 0.0001	
		Duration	−PTX 2.9 s ± 2.3 S.D.	−PTX 5.1 s ± 5.2 S.D.	Mann–Whitney U test	Control *p* = 0.0002	
			+PTX 2.3 s ± 4.3 S.D.	+PTX 3.8 ± 4.8 S.D.	Mann–Whitney U test	*IN:Kcnq2 p* < 0.0001	
							*IN:Kcnq2*: -PTX: *n* = 151; +PTX: *n* = 134 events
Data were obtained from 3 control and 5 *IN:Kcnq2 mice*.							

Figure 5							
Figure			Control	*IN:Kcnq2*	Statistical tests and values		
**5C**	CA3	Amplit.	−D-APV 0.04 Δ*F*/*F* ± 0.02 S.D.	−D-APV 0.06 Δ*F*/*F* ± 0.02 S.D.	Mann–Whitney U test	Control: *p* < 0.0001	Control: -D-APV n = 959; +D-APV: *n* = 565 events
			+D-APV 0.02 Δ*F*/*F* ± 0.007 S.D.	+D-APV 0.03 Δ*F*/*F* ± 0.01 S.D.	Mann–Whitney U test	*IN:Kcnq2*: *p* < 0.0001	
		Duration	−D-APV 0.74 s ± 0.7 S.D.	−D-APV 0.85 s ± 1.7 S.D.	Mann–Whitney U test	Control: *p* < 0.0001	*IN:Kcnq2*: -D-APV n = 412; +D-APV: *n* = 1413events
			+D-APV 0.83 ± 0.6 S.D.	+D-APV 1.1 ± 1.3 S.D.	Mann–Whitney U test	*IN:Kcnq2*: *p* < 0.0001		
	Post. Cortex	Amplit.	−D-APV 0.05 Δ*F*/*F* ± 0.03 S.D.	−D-APV 0.09 Δ*F*/*F* ± 0.06 S.D.	Mann–Whitney U test	Control: *p* < 0.0001	Control: -D-APV n = 457; +D-APV n = 287 events
			+D-APV 0.01 Δ*F*/*F* ± 0.002 S.D.	+D-APV 0.02 Δ*F*/*F* ± 0.02 S.D.	Mann–Whitney U test	*IN:Kcnq2*: *p* < 0.0001	
		Duration	−D-APV 1.7 s ± 1.6 S.D.	−D-APV 2.3 s ± 2.8 S.D.	Mann–Whitney U test	Control: *p* = 0.461	
			+D-APV 1.7 s ± 1.2 S.D.	+D-APV 3.3 ± 2.6 S.D.	Mann–Whitney U test	*IN:Kcnq2*: *p* < 0.0001	
							*IN:Kcnq2*: -D-APV n = 539; +D-APV n = 435 events
	Medial Cortex	Amplit.	−D-APV 0.03 Δ*F*/*F* ± 0.04 S.D.	−D-APV 0.05 Δ*F*/*F* ± 0.04 S.D.	Control: N/A	Control: N/A	Control -D-APV n = 49; +D-APV n = 0 events
			+D-APV N/A	+D-APV 0.01 Δ*F*/*F* ± 0.002 S.D.	*IN:Kcnq2*: Mann–Whitney U test	*IN:Kcnq2*: *p* < 0.0001	
		Duration	−D-APV 0.89 s ± 0.22 S.D.	−D-APV 3.5 ± 2.1 S.D.	Control: N/A	Control: N/A	
			+D-APV N/A	+D-APV 4.1 ± 0.87 S.D.	*IN:Kcnq2*: Mann–Whitney U test	*IN:Kcnq2*: *p* = 0.244	
							*IN:Kcnq2* -D-APV n = 84; +D-APV, n = 5 events;
	Anter. Cortex	Amplit.	−D-APV 0.016 Δ*F*/*F* ± 0.006 S.D.	−D-APV 0.04 Δ*F*/*F* ± 0.05 S.D.	Control: N/A	Control: N/A	Control -D-APV n = 176; +D-APV n = 0 events
			+D-APV N/A	+D-APV 0.02 Δ*F*/*F* ± 0.01 S.D.	*IN:Kcnq2*: Mann–Whitney U test	*IN:Kcnq2*: *p* < 0.0001	
		Duration	−D-APV 1.3 s ± 0.9 S.D.	−D-APV 5.0 s ± 5.1 S.D.	Control: N/A	Control: N/A	
			+D-APV N/A	+D-APV 8.8 ± 3.6 S.D.	*IN:Kcnq2*: Mann–Whitney U test	*IN:Kcnq2*: *p* < 0.0001	
							*IN:Kcnq2*: -D-APV n = 217; +D-APV n = 95 events
Data were obtained from 3 control and 3 *IN:Kcnq2 mice*.							

Figure 6							
P/N/A refers to Picrotoxin/APV/NBQX			Control	*IN:Kcnq2*	Statistical tests and values		
**6C**	CA3	Amplit.	−P/N/A: 0.10 Δ*F*/*F* ± 0.08 S.D.	−P/N/A:0.13 Δ*F*/*F* ± 0.07 S.D.	Mann–Whitney U test	Control: *p* < 0.0001	Control -P/N/A: *n* = 1,083; +P/N/A: *n* = 34 events
			+P/N/A: 0.05 Δ*F*/*F* ± 0.04 S.D.	+P/N/A: Δ*F*/*F* 0.02 ± 0.01 S.D.	Mann–Whitney U test	*IN:Kcnq2 p* < 0.0001	
		Duration	−P/N/A: 0.68 s ± 0.69 S.D.	−P/N/A: 1.76 s ± 2.3 S.D.	Mann–Whitney U test	Control: N/A	
			+P/N/A: 2.8 s ± 4.1 S.D.	+P/N/A: 5.5 s ± 3.3 S.D.		*IN:Kcnq2*: *p* < 0.0001	
							*IN:Kcnq2*: -P/N/A: *n* = 1,630; +P/N/A: *n* = 240 events
	Post. Cortex	Amplit.	0.14 Δ*F*/*F* ± 0.09 S.D.	−P/N/A:0.15 Δ*F*/*F* ± 0.13 S.D.	Mann–Whitney U test	Control: *p* < 0.0001	Control -P/N/A: *n* = 444; +P/N/A: *n* = 3 events
			+P/N/A: 0.02 Δ*F*/*F* ± 0.01 S.D.	+P/N/A: Δ 0.02\u00B0F/F ± 0.01 S.D.		*IN:Kcnq2*: *p* < 0.0001	
		Duration	−P/N/A: 2.6 s ± 2.6 S.D.	−P/N/A: 2.8 s ± 3.0 S.D.	Mann–Whitney U test	Control: *p* < 0.0001	
			+P/N/A: 5.8 s ± 5.6 S.D.	+P/N/A: 8.62 s ± 8.0 S.D.		*IN:Kcnq2*: *p* < 0.0001	
							*IN:Kcnq2*: -P/N/A: *n* = 1,145; +P/N/A: *n* = 177 events	
	Medial Cortex	Amplit.	0.06 Δ*F*/*F* ± 0.07S.D.	−P/N/A:0.02 Δ*F*/*F* ± 0.03 S.D.	Mann–Whitney U test	Control: *p* = 0.26	Control -P/N/A: *n* = 38; +P/N/A: *n* = 2 events
			+P/N/A: 0.014 Δ*F*/*F* ± 0.001 S.D.	+P/N/A: 0.02 Δ*F*/*F* ± 0.02 S.D.		*IN:Kcnq2*: *p* = 0.53	
		Duration	−P/N/A: 1.6 s ± 2.3 S.D.	−P/N/A: 5.2 s ± 6.3 S.D.	Mann–Whitney U test	Control: N/A	
			+P/N/A: 6.1 s ± 0.75 S.D.	+P/N/A: 4.5 s ± 5.2 S.D.		*IN:Kcnq2*: *p* = 0.087	
							*IN:Kcnq2*: -P/N/A: *n* = 424; +P/N/A: *n* = 215 events
	Anter. Cortex	Amplit.	−P/N/A: 0.04 Δ*F*/*F* ± 0.04 S.D.	−P/N/A:0.06 Δ*F*/*F* ± 0.06 S.D.	Mann–Whitney U test	Control: *p* = 0.0004	Control -P/N/A: *n* = 263; +P/N/A: *n* = 5 events
			+P/N/A: 0.013 Δ*F*/*F* ± 0.001 S.D	+P/N/A: 0.02 Δ*F*/*F* ± 0.006 S.D.		*IN:Kcnq2*: *p* < 0.0001	
		Duration	−P/N/A: 1.5 s ± 1.9 S.D.	−P/N/A: 7.1 s ± 4.5 S.D.	Mann–Whitney U test	Control: N/A	
			+P/N/A: 9.2 s ± 2.0 S.D.	+P/N/A: 5.4 s ± 4.0 S.D.		*IN:Kcnq2*: *p* = 0.016	
							*IN:Kcnq2*: -P/N/A: *n* = 404; +P/N/A: *n* = 271 events
Data were obtained from 3 control and 5 *IN:Kcnq2 mice*.							

In the absence of KCNQ2 channels, we found an even greater activity in these subregions reflected as changes in the amplitude, duration, or frequency of calcium events (Figure 1). For instance, in the posterior cortex we found a two-fold increase in the amplitude of the calcium signals (control: 0.053 ± 0.052 Δ*F*/*F*, *n* = 24; *IN:Kcnq2*: 0.14 ± 0.095 Δ*F*/*F*, *n* = 25; *p* < 0.0001, Mann–Whitney U test), whereas in the CA3 region of the hippocampus we observed changes both in the duration of the calcium signal (control: 0.75 ± 0.28 s, *n* = 24,; *IN:Kcnq2*: 0.81 ± 0.26 s, *n* = 26; *p* = 0.0006, Mann–Whitney U test) and its frequency (events/s; control: 0.012 ± 0.012 Hz, *n* = 28; *IN:Kcnq2* 0.019 ± 0.015 Hz, *n* = 26; *p* = 0.0021, Mann–Whitney U test) (Figure 1B). Thus, deletion of *Kcnq2* from GABAergic neurons leads to increases in activity primarily in the hippocampus and posterior cortex, which is comparable to our earlier findings of deleting KCNQ2 channels from excitatory neurons (23).

### Ablation of *Kcnq2* from GABAergic cells increases interneuron population activity across the forebrain in the presence of 8 mM Ko

**Figure 2:**
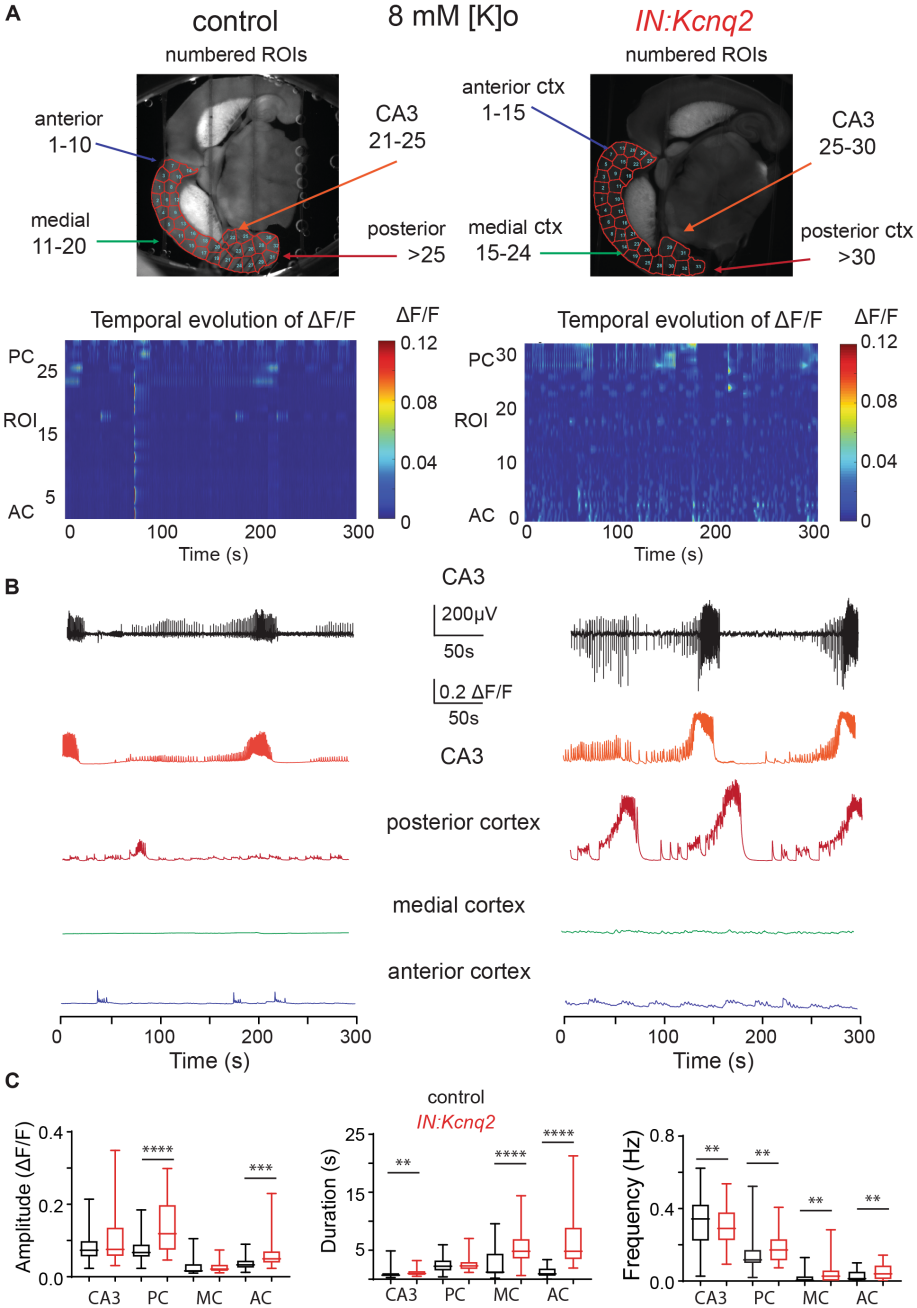
Loss of KCNQ2 from GABAergic cells leads to widespread forebrain hyperexcitability in the presence of 8 mM Ko. **(A)** Top panels: representative examples of horizontals slices expressing GCamp5g from control and *IN:Kcnq2* mice. Each hemisphere was segmented into regions of interest (ROI). Bottom panels: 2D plots showing the changes in the calcium signal measured as Δ*F*/*F* for the different ROIs during the 5 min recording period. The ROI numbering represents the various segmented areas with lower values towards the anterior cortex and higher values towards the posterior cortex and the CA3 area of the hippocampus. **(B)** Top panels: LFPs recorded in parallel to the calcium signal from the CA3 region of the hippocampus. Bottom panels: Temporal evolution of Δ*F*/*F* across multiple ROIs. ROIs represent different forebrain anatomical areas (CA3 region of the hippocampus, posterior, medial, and anterior cortex). **(C)** Summary graphs showing the effect of *Kcnq2* ablation to the amplitude (Δ*F*/*F*), duration(s), and frequency (Hz; events/s) as box plots of the calcium events for different anatomic regions. MC, medial cortex; PC, posterior cortex; AC, anterior cortex. Data are presented as box-and-whisker plots (box shows the 25th and 75th percentiles, whereas the whiskers show the minimum and maximum values). Detailed data and statistics could be found in Table 1; Figure 2C section.

The low basal activity in the neocortex in 2.5 mM extracellular potassium prevented us from determining whether KCNQ2 channels also play a role in the neocortex, a region that highly expresses KCNQ2 channels across development. This is because spontaneous neocortical activity in slices at these ages is sparse (45). Thus, we examined the effects of ablating KCNQ2 channels from GABAergic cells in slices superfused with 8 mM Ko. Increasing the extracellular potassium depolarizes neurons as it shifts the Nernst equilibrium potassium potential to more positive values, leading to increased neuronal excitability. Despite the change in the extracellular potassium concentration, calcium activity was still restricted to the hippocampal formation in our control slices (Figure 2A). However, in slices deficient of KCNQ2 channels, we detected activity across the hemisphere with prominent activity both at the hippocampal formation and the neocortex. This is clearly illustrated in the two-dimensional plot in Figure 2A. Notice that in the absence of KCNQ2 channels fluorescence signal is apparent across the hemisphere.

To quantify the impact of KCNQ2 deletion from GABAergic interneurons, we divided our hemispheres into different subregions and determined the amplitude, duration, and frequency of the calcium events (Figure 2B). With respect to the amplitude, we found that *Kcnq2* ablation increased calcium activity primarily in the posterior and anterior cortex, whereas the duration of the calcium signals was increased in all regions except the posterior cortex. The amplitude and duration of the calcium signals were a function of the number and duration of neurons active at any given moment. Thus, the aforementioned changes suggest that *Kcnq2* deletion increases the population activity of interneurons across the neocortex. This would be consistent with an increased excitability of interneurons, making them more prone to firing action potentials following incoming activity and further explaining the increase in the frequency of calcium events in the neocortex (Figures 2B,C).

**Figure 3:**
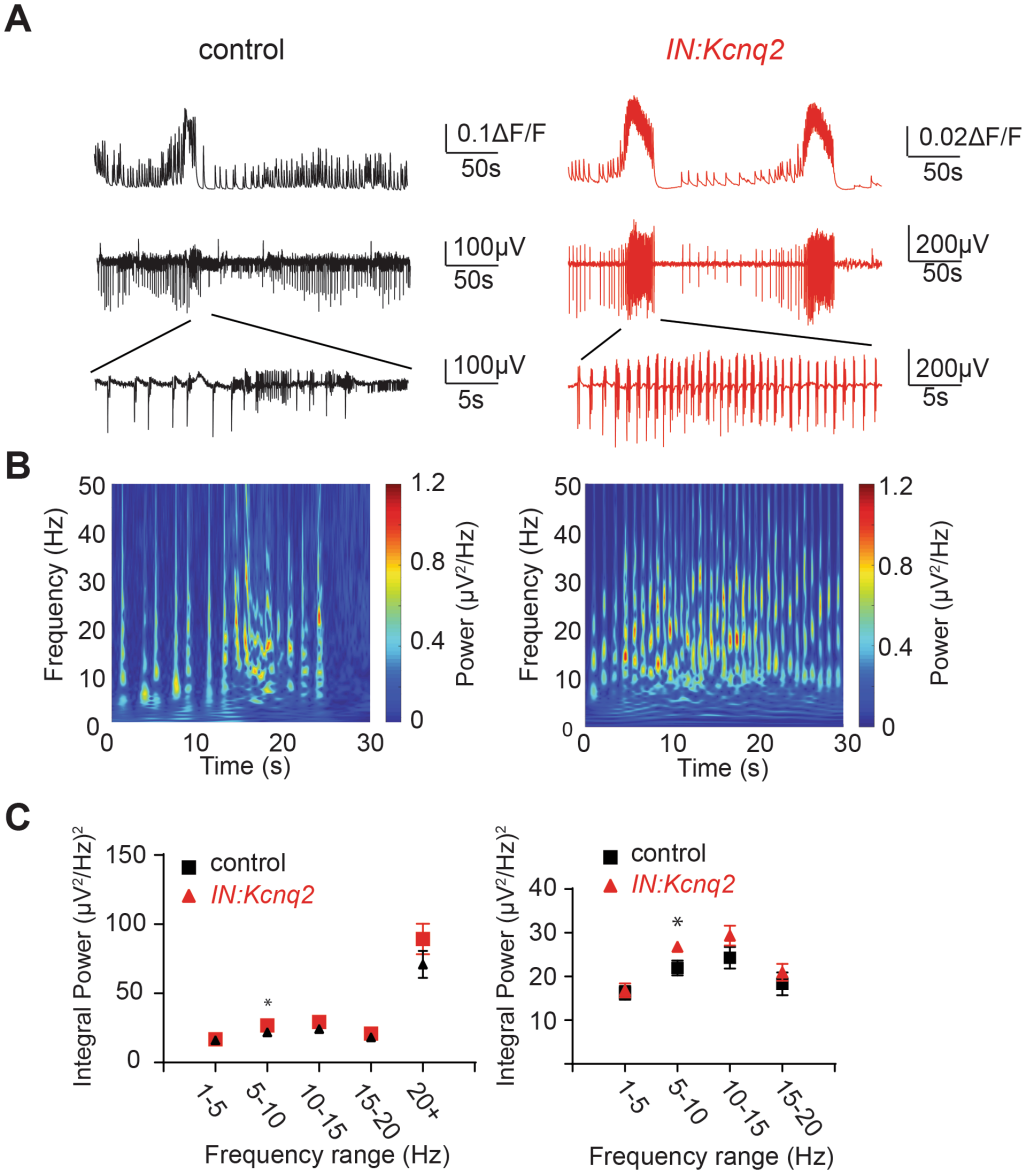
Deletion of *Kcnq2* from GABAergic cells had modest effects on the local field potential (LFP) measured in the CA3 of the hippocampus. **(A)** Top panels: calcium signal Δ*F*/*F* from the CA3 hippocampus in the presence of 8 mM Ko. Middle panels: LFP from the same region as in the top panels recorded in parallel. Notice that the close correspondence of the LFP and the calcium signal. Bottom panels: zoom-in on the LFP signals showing 30 s of recording of bursting activity. **(B)** Time-frequency scalogram of the LFP zoom-in signals reported in **(A)** (bottom panels). The color scale indicates the power spectrum density (*μV*^2^/Hz). Note that the power density is mainly concentrated between 5 Hz and 30 Hz throughout the duration of the burst. **(C)** Left panel: spectral power (*μV*^2^) calculated in five frequency ranges. Right panel: same data as in the left panel with frequency ranges up to 20 Hz. Note the significant increase in power in the 5–10 Hz. Power values are obtained by integration of the scalogram over time (0–30 s) and frequency range. Data are presented as mean and S.E.M. Detailed data and statistics for these graphs are reported under Table 1; Figure 3C section.

In parallel to our calcium measurements, we recorded the LFP from the CA3 region of the hippocampus. With an elevated potassium concentration (8 mM Ko), we routinely observed robust LFP activity independent of the presence or absence of KCNQ2 channels. We also noticed that the LFP activity was comprised of continuous events with intermittent bursts lasting ~30 s (Figure 2B), with this bursting being independent of the presence of KCNQ2 channels. Next, we assessed the spectral content of these bursts (Figure 3A) and we found that, while bursts had power spectra spanning across the theta (3–7 Hz), alpha (8–14 Hz), and beta (15–30 Hz) frequencies (Figure 3B), oscillations in the range 5–10 Hz (i.e., upper theta/low alpha frequencies) showed a significant increase (Figure 3C). The 5–10 Hz frequency band likely reflects oscillations in the alpha frequency range, which have been previously associated with increased fast spiking interneuron activity.

### Blocking GABAA receptors increases GABAergic interneuron network activity

GABAergic interneurons release GABA and activate GABAA receptors, which in turn inhibit or facilitate activity based on the equilibrium membrane potential of chloride ions. At our developmental time point (P4–P7), GABA is primarily inhibitory in the neocortex (45, 46) whereas in the hippocampus it still transitions from excitatory to inhibitory as recently reported by Murata et al., i.e., excitatory at P3 and inhibitory by P7 (46). Additionally, multiple groups have shown that in addition to phasic GABA release, GABA is found tonically activating extracellular GABAA receptors (47). We previously found that application of picrotoxin, a GABAA receptor inhibitor that blocks both synaptic and extrasynaptic GABAA receptors, increases excitability in brain slices of 1 week-old animals (23). Thus, we hypothesized that eliminating GABAA receptor activity would also allow for greater interneuron excitability due to unabated excitatory network activity.

**Figure 4:**
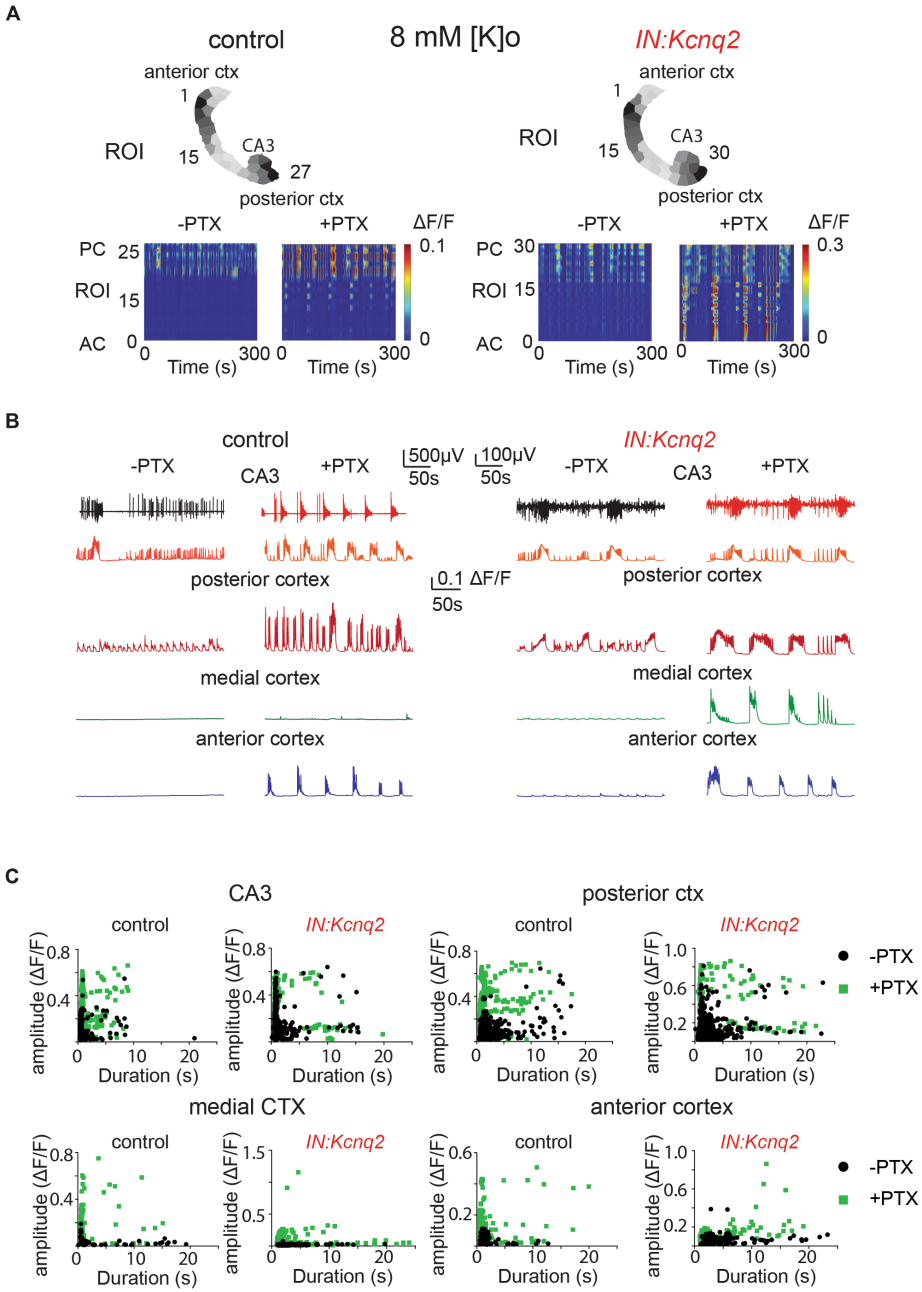
GABAA receptors limit activity from both wild-type and *Kcnq2*-null GABAergic neonatal brain slices. **(A)** Two examples of acute slices from control and *IN:Kcnq2* mice (left and right column, respectively) before (−PTX) and after (+PTX) the application of 50 μM picrotoxin (PTX) to block synaptic and extrasynaptic GABAA receptors. Top panels: horizontal slices segmented into regions of interest (ROI). Bottom panels: changes in the Δ*F*/*F* as a function of time for the different ROIs. The numbering corresponds to the segmented area in the top panels, with lower values towards the anterior cortex and higher values at the posterior cortex and CA3 area of the hippocampus. **(B)** Temporal evolution of the LFP (top lines) and the Δ*F*/*F* signals (bottom lines) for different anatomical regions (CA3, posterior cortex, medial cortex, and anterior cortex) before and after application of 50 μM PTX. **(C)** Scatter plots showing the effect in amplitude and duration for the different anatomical regions (CA3, posterior cortex, medial cortex, and anterior cortex) before and after application of 50 μM PTX. Details on the statistical analysis and number of replicates for this figure are found under Table 1; Figure 4C section.

To investigate the effects of GABAA receptor inhibition on the interneuron network activity, we first applied 50 μM picrotoxin in slices from control animals (Figure 4A). Blocking GABAA receptors had several effects. First, calcium activity was no longer restricted to the hippocampal formation, but instead was present across multiple regions. This was especially evident in the anterior cortex, where the barely detectable initial activity levels increased to large calcium events in the presence of picrotoxin. Second, the activity became more periodic and synchronous, with bursting activity followed by quiescence periods (Figures 4A,B). Third, the amplitude and duration of the calcium events became larger and longer, likely reflecting greater recruitment of interneurons in the network (Figures 4B,C).

Next, we investigated the effect of blocking inhibition in the IN:Kcnq2 slices. Similar to the slices from control animals, we found that activity was synchronous and present across all regions in the IN:Kcnq2 slices (Figures 4A,B). We observed robust events even in the medial cortex, a region typically showing very low-level activity in slices from control animals (Figures 4B,C). Additionally, we observed large calcium events independent of whether their duration was brief or long lasting (Figure 4C). Overall, the loss of KCNQ2 channels from interneurons further amplified the pro-excitatory effects of picrotoxin. Together, our data suggest that the main role of GABAergic interneuron activity is inhibitory at the network population level.

### Blocking NMDA receptors dampens interneuron network activity

**Figure 5:**
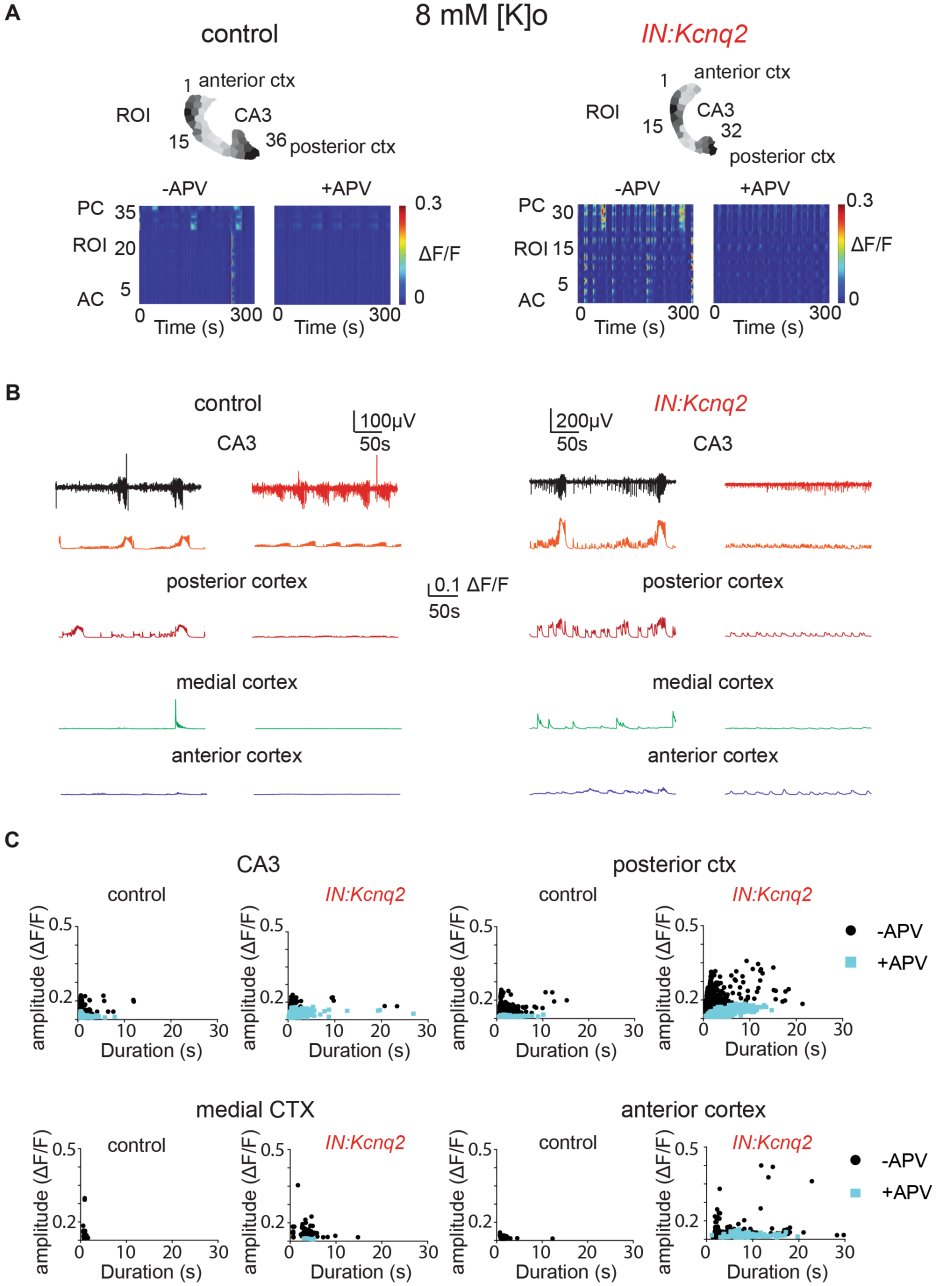
GABAergic population excitability is partly driven by NMDA receptors. **(A)** Two examples of acute slices from control and *IN:Kcnq2* mice (left and right column, respectively) before (−APV) and after (+APV) the application of 25 μM D-APV in the presence of 8 mM Ko. Top panels: horizontal slices segmented into regions of interest (ROI). Bottom panels: changes in the Δ*F*/*F* as a function of time for the different ROIs. The numbering corresponds to the segmented area in the top panels, with lower values towards the anterior cortex and higher values at the posterior cortex and CA3 area of the hippocampus. **(B)** Temporal evolution of the LFP (top lines) and the Δ*F*/*F* signals (bottom lines) for different anatomical regions (CA3, posterior cortex, medial cortex, and anterior cortex) before and after application of 25 μM D-APV. **(C)** Scatter plots showing the effect in amplitude and duration for the different anatomical regions (CA3, posterior cortex, medial cortex, and anterior cortex) before and after application of 25 μM D-APV. Additional details on the statistical analysis and number of replicates for this figure are found under Table 1; Figure 5C section.

Multiple groups have shown that network population events in slices depend on ongoing excitatory network activity (43, 44, 48, 49). In neonatal mice, NMDA receptors have been proposed to have a strong excitatory role (43, 50). As our recording conditions included elevated potassium concentrations, which would make it easier to relieve the magnesium block, the role of the NMDA receptors might have been enhanced. Indeed, we found that in slices from control animals, application of the competitive NMDA receptor antagonist APV (25 μM) substantially reduced the LFP, the calcium event frequency, and on many occasions the amplitude and duration of the calcium events (Figure 5). We observed similar effects in slices from IN:Kcnq2 mice (Figure 5A). However, one notable exception was in the anterior cortex, where application of APV in IN:Kcnq2 slices reduced the frequency of the events but had very weak effects on the amplitude and duration of the calcium events (Figures 5B,C).

**Figure 6:**
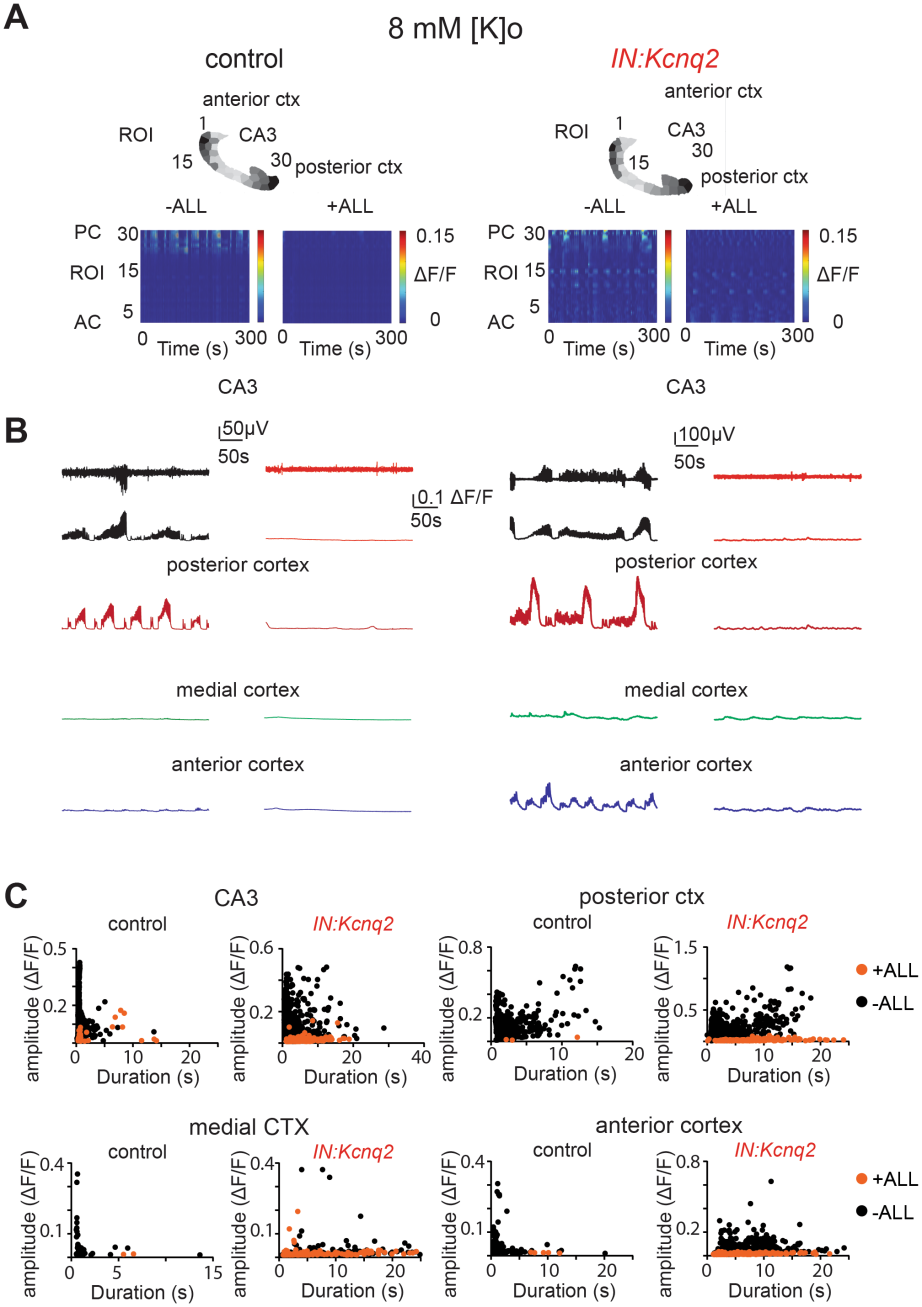
Fast synaptic transmission receptor activity drives calcium population changes in GABAergic neurons deficient of KCNQ2 channels. **(A)** Two examples of acute slices from control and *IN:Kcnq2* mice (left and right column, respectively) before (−ALL) and after (+ALL) the application of 50 μM PTX, 25 μM D-APV, and 25 μM NBQX. Top panels: horizontal slices segmented into regions of interest (ROI). Bottom panels: changes in the Δ*F*/*F* as a function of time for the different ROIs. The numbering corresponds to the segmented area in the top panels, with lower values towards the anterior cortex and higher values at the posterior cortex and CA3 area of the hippocampus. **(B)** Temporal evolution of the LFP (top lines) and the Δ*F*/*F* signals (bottom lines) for different anatomical regions (CA3, posterior cortex, medial cortex, and anterior cortex) before and after application of 50 μM PTX, 50 μM D-APV, and 25 μM NBQX. **(C)** Scatter plots showing the effect in amplitude and duration for the different anatomical regions (CA3, posterior cortex, medial cortex, and anterior cortex) before and after application of 50 μM PTX, 50 μM D-APV, and 25 μM NBQX. Additional details on the statistical analysis and number of replicates for this figure are found under Table 1; Figure 6C section.

Not surprisingly, simultaneous application of synaptic blockers for AMPA, NMDA, and GABAA receptors almost entirely eliminated calcium activity in control slices (Figure 6A). Similarly, we found a very strong reduction in interneuron activity in slices from IN:Kcnq2 mice upon application of all three synaptic blockers. We did notice some remaining low-level activity in the medial and anterior cortex (Figures 6B,C; Table 1); however, this activity was barely above background levels. Thus, GABAergic interneuron activity in neonatal slices is driven primarily by fast excitatory transmission, and loss of KCNQ2 channels from interneurons increases their population activity.

## Discussion

In this study, we defined the role of KCNQ2 channels in the interneuron network excitability of the forebrain. Currently, very few studies have focused on GABAergic interneurons and the impact of KCNQ2 channels on their properties. By using conditional *Kcnq2* knockout mice, we found that deletion of *Kcnq2* from GABAergic interneurons early in life increases their excitability across the hippocampal formation and neocortex. Thus, our work suggests that KCNQ2 channel variants alter not only the excitability properties of immature excitatory cells, but also the properties of immature GABAergic cells.

### KCNQ2 channels and GABAergic interneurons

KCNQ2 channels are a core component of the M-current (51, 52), a slowly activating non-inactivating voltage-gated potassium channel. The M-current’s main function is to prevent excessive firing (53). Thus, following strong activity, KCNQ2 channels and the M-current are activated, leading to spike frequency adaptation. This quiescence period is most typically associated with excitatory cells, and in particular pyramidal neurons (54). Consequently, for many years, the roles of the M-current and KCNQ2 channels in interneurons have been ignored. Despite this, a few studies using pharmacology have shown that a KCNQ-mediated current is present in interneurons, including somatostatin and parvalbumin GABAergic cells of the hippocampus (33, 35, 55). We and others have also shown that deletion of KCNQ2 channels from parvalbumin interneurons increases their firing activity, leading to elevated spontaneous inhibitory transmission (35, 36). Our study reveals that deletion of KCNQ2 channels from GABAergic neurons has a significant effect on the posterior cortex, which includes the entorhinal cortex, as well as on the CA3 area of the hippocampus. Previous studies have shown that the entorhinal cortex displays prominent spontaneous activity early in life (43, 44), potentially due to its high level of interconnectivity (56), although the exact mechanism remains unclear. The absence of KCNQ2 channels from GABAergic neurons is likely to amplify the propensity for spontaneous activity in these two regions, suggesting that the loss of KCNQ2 activity may have a more pronounced effect on these regions than on other cortical regions such as the medial and anterior cortex. Although the activity in these regions also increased in the absence of KCNQ2 channels from GABAergic neurons, the effect was much weaker than in the posterior cortex and hippocampal CA3 area.

A limitation of previous studies was the age of the animals. Most prior studies on KCNQ2 channels and interneuron activity were performed in juvenile or adult mice (33, 35, 36, 55). However, KCNQ2 channels are already expressed prior to birth, with their levels increasing as the animals further develop (18). Considering the key role of GABAergic transmission in the development of neuronal networks (48, 57), we sought to examine whether KCNQ2 channels also alter GABAergic interneuron activity in immature neurons. We found that, indeed, loss of KCNQ2 channel activity leads to elevated interneuron activity across the forebrain. Therefore, we suggest that KCNQ2 channels act as a brake early in development, limiting the recruitment of interneurons. This was evident in experiments in which we blocked GABAergic transmission.

Consistent with some recent papers (58, 59), we found that application of the GABAA receptor antagonist picrotoxin increases interneuron network excitability in both control and *Kcnq2* GABAergic null neurons. This result is consistent with earlier and more recent findings that GABAA receptors are inhibitory at the network level within the first week of rodent life (46, 58–60). Although several studies have shown that GABA acts to depolarize neurons, at the network level its effects are likely inhibitory (59). This could be because tonic GABAA receptor activity likely acts as a shunt, clamping the resting potential of neurons to values that are depolarized yet subthreshold to action potential generation.

### Implication to KCNQ2 loss-of-function variants

A major development over the last 10 years has been the recognition that KCNQ2 loss-of-function variants can lead to severe DEE (4, 5). Our current and prior work suggest that KCNQ2 loss of function would impair the function of both excitatory and GABAergic neurons. An unanswered question is whether loss of KCNQ2 channel activity from interneurons will further promote seizures or will dampen overall activity. In our previous study, deletion of *Kcnq2* solely from interneurons led to increased excitatory transmission due to homeostatic changes (36). However, in constitutive *Kcnq2* loss-of-function mice, pyramidal neuron excitability would already be elevated; thus, additional homeostatic changes should not lead to any further increase in neuronal excitability. We predict that loss of *Kcnq2* from interneurons might instead have a protective role. Indeed, when we crossed our IN:Kcnq2 mice to Pyr:Kcnq2 mice (forebrain excitatory neuron *Kcnq2* null mice), we found that the mice survived longer than the Pyr:Kcnq2 mice (data not shown). In contrast, pharmacological activation of KCNQ2 channels using KCNQ activators such as retigabine (61) will dampen excitability of GABAergic interneurons, which might decrease their ability to dampen overall network excitability. Indeed, a recent study showed the deleting KCNQ2 channels from parvalbumin interneurons allowed retigabine to have stronger anti-convulsant effects (35).

### Limitations of our study

A limitation of this study is the lack of determination of the impact of KCNQ2 channels on interneuron neuronal physiology in different subregions at a single-cell level. For instance, we do not know whether all types of interneurons at this early stage of life depend on KCNQ2 channel activity or whether only a subset depends on them. We also do not know whether ablation of KCNQ2 channels from interneurons alters the properties of the excitatory cells or whether the synaptic drive to interneurons is affected. Lastly, it is possible that some of our effects might not solely be due to loss of KCNQ2 channel activity. For instance, KCNQ2 channels form heteromers with KCNQ3 and KCNQ5 channels (62). Thus, deletion of KCNQ2 might also alter the protein levels of KCNQ channels in general (37), further amplifying the observed phenotype.

## Conclusion

Deleting KCNQ2 channels from GABAergic cells leads to increased GABAergic interneuron population activity in the neonatal forebrain. Our study fills a significant gap in our knowledge by demonstrating that KCNQ2 channels are important regulators of not only immature excitatory neurons networks but also GABAergic interneuron networks. Thus, KCNQ2 pathogenic variants or drugs targeting KCNQ2 channels early in life will modify the activity of both glutamatergic and GABAergic cells.

## Data availability statement

The original contributions presented in the study are included in the article/Supplementary material. Further inquiries can be directed to the corresponding author.

## Ethics statement

The animal study was reviewed and approved by the University of Connecticut Institutional Animal Care and Use Committee (IACUC).

## Author contributions

BH, SS, and AT: designed research and wrote the paper. BH: performed research. BH and SS: analyzed data. All authors contributed to the article and approved the submitted version.

## Funding

This work was supported by National Institutes of Health Grants R01 NS101596, NS108874 (to AT), and NSF CAREER Award 1845348 (to SS).

## Conflict of interest

The authors declare that the research was conducted in the absence of any commercial or financial relationships that could be construed as a potential conflict of interest.

## Publisher’s note

All claims expressed in this article are solely those of the authors and do not necessarily represent those of their affiliated organizations, or those of the publisher, the editors and the reviewers. Any product that may be evaluated in this article, or claim that may be made by its manufacturer, is not guaranteed or endorsed by the publisher.

## Footnotes

^1^https://imagej.net/software/fiji/
